# Medical and Physical Intelligence

**Published:** 1804-11-01

**Authors:** 


### Medical and Physical Intelligence.

### PLAN of the ROYAL SUSSEX JENNERIAN INSTITUTION.

I. That as his Royal Highness the Prince of Wales has been graciously pleased to honour this institution by his patronage, it be denominated "The Royal Sussex Jennerian Institution," for the Extermination of the Small-pox.

II. An annual meeting of this Institution is to be held on the day appointed for the meeting of the Agricultural Society at Lewes.

III. A subscription of five guineas at one payment, or of one guinea annually, constitutes a Governor; and a Governor is entitled to recommend patients for the benefit of this institution.

IV. A Board of Directors shall be formed, consisting of a President, Vice-President, and 24 Members, who are Governors of this Institution, and not of the Medical Profession, five of whom shall be a quorum; one third of this Board shall go out annually, and the vacancies shall be supplied by lot, from the list of the Governors. They shall meet annually, at the above mentioned time, or oftener if expedient, to take into consideration the general interests of the Society, and to receive the reports of the Medical Council. This Board shall be empowered to frame laws and regulations, by which the Institution shall be governed; and they shall order the Trustees to pay such monies as shall appear to have been expended in forwarding the views of the Institution.

V. Trustees shall be appointed by the Board of Directors, who shall manage the pecuniary concerns of this Society, and order the Treasurer to make the necessary payments; they shall examine the Treasurer's accounts, previous to their being laid before the general meeting, or oftener if deemed expedient:—they shall be empowered to invest in their own names, for the use of this Society, such sums in the public funds, as shall be unappropriated by the Board of Directors.

VI. Mr. Whitfield is appointed Treasurer, who shall be empowered to receive subscriptions for forwarding this Institution; he shall keep a regular account of receipts and disbursements, and lay such accounts before the Trustees at least once a year, and oftener if deemed necessary; and he shall pay monies upon the order of the Trustees, giving in writing.

VII. A General Court may be convened by five Governors, expressing their wish in writing to the Secretary of the Institution, and Medical Council; who shall call the same by notice in the Lewes paper, at least one week before such meeting be appointed to be held.

VIII. Parish Officers shall be requested and enjoined to have the paupers of their respective parishes immediately inoculated with Cow-pock, and the medical Gentlemen employed, are requested to keep a register of such patients as they may inoculate.

IX. A Medical Council shall be appointed, to consist of a President, Vice President and 36 Members, one-third of whom to go out out annually in rotation, and the first 12 by ballot. No President or Vice President shall be chosen two succeeding years. This Council shall name 24 Medical Gentlemen, 12 of whom shall be chosen by ballot, to succeed to the vacancies, and from the new Council the President and Vice President shall be also appointed by majority of votes; and in case of an equal number of votes, the President of the last year shall decide; and this election is to take place at Brighton, the 17th of May in each year, being the birth-day of Dr. Jenner.

X. The business of the Medical Council shall be to superintend the Medical concerns of the Society; to appoint stations in the County for gratuitous Inoculation of the Cow-pock, and at which supplies of Cow-pock matter may be constantly kept; to give instruction for Inoculation, and to make a report of the progress of the same to the Board of Directors.

XI. The Medical Council shall meet the first Thursday of every month, or oftener if deemed necessary, five to be a quorum; and these meetings shall be alternately at Brighton and Lewes.

XII. The stations for gratuitous Inoculation shall be the following:—Chichester, Arundel, Midhurst, Petworth, Worthing, Steyning, Horsham, Brighton, Lewes, East Grinstead, Seaford, East Bourne, Battle, Tunbridge Wells, Hastings, and Rye.

XIII. At each station, a Surgeon shall attend two days in every week, viz. on the market day, and the fourth day after, between the hours of nine and ten o'clock, to inoculate gratis such persons as apply, and appear to be proper objects; no person to be inoculated who does not promise to attend on the days he is desired.

XIV. The Surgeons in each station, shall, in rotation, continue to perform this duty for three months, and it shall be conducted at the house of the acting Surgeon, who shall keep a register, and whose business shall also be to preserve virus according to the directions of the Medical Council; and to distribute it to such Surgeons in the County as may apply for the same; a list of whose names shall be preserved.

XV. The Physicians in each station, by rotation, are to attend gratis at such appointed days for the purpose of consultation on any doubtful cases that may arise; or on any other circumstance relating to this subject, that may require their attention; and they are to keep notes of remarkable occurrences.

XVI. The Secretary's duty shall be to attend Medical and General Meetings; he shall keep Minutes of their proceedings, and prepare them to be laid before the General Meeting. All letters relative to the concerns of the Society, (except for vaccine virus), shall be addressed to him, who shall lay them before the Medical Council. Registers kept, as before directed, shall be transmitted to the Secretary, at least one fortnight before the General Meeting, and he shall arrange and prepare such to be laid before the Medical Council, one week before the General Meeting.

XVII. The Medical Council is empowered to appoint any other stations for Inoculation they may think necessary, and to form any other other regulations that may appear to them to forward the views of the Institution.

XVIII. The Medical Council shall occasionally communicate with, and at least once in each year transmit to the Secretary of the ROYAL JENNERIAN INSTITUTION in London, a general statement of the progress of this Institution; and an accurate account of the number of persons who shall be vaccinated under its direction.

---

Mr. EDLIN, in a letter to the Editors, dated Uxbridge, Oct. 8, 1804, says, "I should be obliged to you to have the goodness to announce in the next number of the Medical and Physical Journal, that I have in the press a Treatise on the Art of Bread-making; great part of it is worked off, and Mr. Hood informs me that it will be ready for publication some time in November. The intention of the work is to concentrate into one point of view every thing that is at present known respecting the manufacture of bread, in order that the knowledge of an intricate and interesting subject, which has hitherto been very imperfectly explored, might be diffused through every class of society. And to accommodate it to the inferior orders of men, it will be printed in as cheap and compact a form as the quantity of letter-press will allow.

The subjects that are proposed to be discussed are divided into the following heads:

1st. The Natural History and Cultivation of Wheat. 2d. The Mealing Trade, including the grinding of Wheat and dressing it into Flour. 3d. The Analysis of Wheat Flour. 4th. The Analysis of Yeast. 5th. The Theory of Fermentation in Bread. 6th. The Preparation of Bread, including a compleat Account of the Baker's Mode of making Bread and Rolls. 7th. The Substitutes for Wheaten Bread. 8th. The Preparation and Preservation of Yeast. 9th. The Structure of a Bakehouse. 10th. The Assize Laws and Manner of regulating the Price of Bread.

---

Dr. BEDDOES, in a letter to Dr. Bradley, says, that various interruptions prevented him in the first place from forwarding the concluding Observations on the Influenza; and that latterly he expected to be able to obtain from the Continent, some valuable intelligence as to its course; a part of its history so necessary towards judging of its contagious or non-contagious nature.

He adds, that immediately on the close of the present year will be put to press an ample Report of the Proceedings at the Preventive Medical Institution. This institution would enable him immediately to give full employment to an additional medical assistant, who, besides being worthy of recommendation on account of his diligence and moral qualities, should be well acquainted with the common doctrines and practices of medicine. Dr. B. conceives, that among medical students who have gone through their elementary instructions, and are not immediately disposed to settle, there must be many, to whom the situation would be highly advantageous, especially, as to ample experience it would add opportunities of human and comparative anatomy, physiological researches, &c.

